# Supplementary material for: The burden and dynamics of hospital-acquired SARS-CoV-2 in England
Source: Nature. 2023 Oct 18;623(7985):132–8. doi: 10.1038/s41586-023-06634-z (PMC10620085; doi:10.1038/s41586-023-06634-z)
Supplement: Supplementary file 1 — Supplementary Methods and Results. The Methods section includes equations for the compartmental model coupling hospital and community dynamics and tables describing model variables and their initial values (Supplementary Table 1) and model parameters (Supplementary Table 2) including values considered. This section also includes methods for the comparison of seroprevalence in healthcare workers versus the community. The Results section includes results from several sensitivity analyses for quantifying the number of hospital-acquired infections and full results from the negative binomial auto-regressive models (Supplementary Tables 3–18 and Supplementary Fig. 1, which shows the estimated spline function from model P1.1.1.tv). [file 41586_2023_6634_MOESM1_ESM.pdf]

nature portfolio

https://doi.org/10.1038/s41586-023-06634-z

Supplementary information

The burden and dynamics of hospital-acquired SARS-CoV-2 in England

In the format provided by the authors and unedited

Supplementary Information (SI)

1 SUPPLEMENTARY METHODS **2**

1.1 Contents of this section **2**

1.2 Equations for the compartmental model coupling hospital and community dynamics **3**
Table S1: Model variables 5
Table S2: Model Parameters 9

1.3 Seroprevalence in healthcare-workers versus the community: international comparisons **18**

2 SUPPLEMENTARY RESULTS **19**

2.1 Quantifying the number of hospital-acquired infections: sensitivity analyses. **19**

2.2 Full results from analysis with negative binomial auto-regressive models **20**

Outcome 1: Indeterminate, probable and definite healthcare associated infections. **20**
Table S3: Model P1.1.1 20
Table S4: Model P1.1.0 21
Table S5: Model P1.0.0 21

Outcome 2: Probable and definite healthcare associated infections. **22**
Table S7: Model P1.1.0 23
Table S8: Model P1.0.0 24

Outcome 3: Definite healthcare associated infections. **24**
Table S9: Model P1.1.1 24
Table S10: Model P1.1.0 25
Table S11: Model P1.0.0 26

Outcome 4: HCW infections. **26**
Table S12: Model P1.1.1 26
Table S13: Model P1.1.0 27
Table S14: Model P1.0.0 27

Outcome 5: Negative control outcome - admissions of patients with confirmed community-acquired Covid-19 infections. **28**
Table S15: Model P1.1.1 28
Table S16: Model P1.1.0 29
Table S17: Model P1.0.0 29

Sensitivity analysis with outcome 2 (probable and definite healthcare associated infections) and a degree 3 spline model allowing for time-varying changes in the number of hospital-acquired infections not accounted for by the covariates. **30**
Table S18 Model P1.1.1.tv 30
Figure S1 | Estimated spline function from Model P1.1.1.tv. 31

2.3 Generation and analysis of synthetic data **31**

2.4 Vaccine coverage in patients **34**

2.5 Seroprevalence in HCWs versus the community: international comparisons **34**

1 Supplementary methods

1.1 Contents of this section

Equations for the compartmental model coupling hospital and community dynamics are given in section 1.2 below. The variables used in this model are defined in table S1 below while model parameters and values considered are defined in table S2 below. Section 1.3 describes methods for international comparisons of seroprevalence in healthcare-workers versus the community.

.

1.2 Equations for the compartmental model coupling hospital and community dynamics

1 a) For the hospitalised population

$$\frac{\mathrm{d}S_H}{\mathrm{d}t} = \frac{-\beta_{H,H}\, S_H(I1_H + I2_H) - \beta_{H',H}\, S_H\, I'_H - \beta_{HCW,H}\, S_H\, I1_{HCW}}{N_H} - \mu S_H + \alpha S_C$$

$$\frac{\mathrm{d}E1_H}{\mathrm{d}t} = \frac{\beta_{H,H}\, S_H(I1_H + I2_H) + \beta_{H',H}\, S_H\, I'_H + \beta_{HCW,H}\, S_H\, I1_{HCW}}{N_H} - \gamma_1 E1_H - \mu E1_H + \alpha E1_C$$

$$\frac{\mathrm{d}E2_H}{\mathrm{d}t} = \gamma_1 E1_H - \gamma_2 E2_H - \mu E2_H + \alpha E2_C$$

$$\frac{\mathrm{d}I1_H}{\mathrm{d}t} = \gamma_2 E2_H - \rho_1 I1_H - \mu I1_H + \alpha I1_C$$

$$\frac{\mathrm{d}I2_H}{\mathrm{d}t} = \rho_1 I1_H - \rho_2 I1_H - \mu I2_H + \alpha I2_C$$

$$\frac{\mathrm{d}I'_H}{\mathrm{d}t} = \pi_H\, \rho_2\, I2_H - \rho_3 I'_H - \mu' I'_H + \alpha' I'_C$$

$$\frac{\mathrm{d}R_H}{\mathrm{d}t} = (1 - \pi_H)\, \rho_2\, I2_H + \rho_3 I'_H - \mu R_H + \alpha R_C$$

b) For the community population

$$\frac{\mathrm{d}S_C}{\mathrm{d}t} = \frac{-\phi\,\beta_{C,C}\,S_C(I1_C + I2_C) - \phi\,\beta'_{C,C}\,S_C I'_C - \beta_{HCW,C}\,S_C\,I1_{HCW}}{N_C} + \mu S_H - \alpha S_C$$

$$\frac{\mathrm{d}E1_C}{\mathrm{d}t} = \frac{\phi\,\beta_{C,C}\,S_C(I1_C + I2_C) + \phi\,\beta'_{C,C}\,S_C I'_C + \beta_{HCW,C}\,S_C\,I1_{HCW}}{N_C} - \gamma_1 E1_C + \mu E1_H - \alpha E1_C$$

$$\frac{\mathrm{d}E2_C}{\mathrm{d}t} = \gamma_1\,E1_C - \gamma_2\,E2_C + \mu\,E2_H - \alpha\,E2_C$$

$$\frac{\mathrm{d}I1_C}{\mathrm{d}t} = \gamma_2\,E2_C - \rho_1\,I1_C + \mu\,I1_H - \alpha\,I1_C$$

$$\frac{\mathrm{d}I2_C}{\mathrm{d}t} = \rho_1\,I1_C - \rho_2\,I2_C + \mu\,I2_H - \alpha\,I2_C$$

$$\frac{\mathrm{d}I'_C}{\mathrm{d}t} = \pi_C\,\rho_2\,I2_C - \rho_3 I'_C + \mu'\,I'_H - \alpha'\,I'_C$$

$$\frac{\mathrm{d}R_C}{\mathrm{d}t} = (1 - \pi_C)\,\rho_2\,I2_C + \rho_3 I'_C + \mu R_H - \alpha R_C$$

c) For HCWs

$$\frac{\mathrm{d}S_{HCW}}{\mathrm{d}t} = \frac{-\beta_{H,HCW}\,S_{HCW}(I1_H + I2_H) - \beta'_{H,HCW}\,S_{HCW} I'_H - \beta_{HCW,HCW}\,S_{HCW}(I1_{HCW} + I2_{HCW}) - \phi\,\beta_{C,HCW}\,S_{HCW}(I1_C + I2_C)}{N_{HCW}}$$

$$\frac{\mathrm{d}E1_{HCW}}{\mathrm{d}t} = \frac{\beta_{H,HCW}\,S_{HCW}(I1_H + I2_H) + \beta'_{H,HCW}\,S_{HCW} I'_H + \beta_{HCW,HCW}\,S_{HCW}(I1_{HCW} + I2_{HCW}) + \phi\,\beta_{C,HCW}\,S_{HCW}(I1_C + I2_C)}{N_{HCW}} - \gamma_1 E1_{HCW}$$

$$\frac{\mathrm{d}E2_{HCW}}{\mathrm{d}t} = \gamma_1\,E1_{HCW} - \gamma_2\,E2_{HCW}$$

$$\frac{\mathrm{d}I1_{HCW}}{\mathrm{d}t} = \gamma_2\, E2_{HCW} - \rho_1\, I1_{HCW}$$

$$\frac{\mathrm{d}I2_{HCW}}{\mathrm{d}t} = \rho_1\, I1_{HCW} - \rho_2\, I2_{HCW}$$

$$\frac{\mathrm{d}I'_{HCW}}{\mathrm{d}t} = \pi_{HCW}\, \rho_2\, I2_{HCW} - \rho_3\, I'_{HCW}$$

$$\frac{\mathrm{d}R_{HCW}}{\mathrm{d}t} = (1 - \pi_{HCW})\rho_2\, I2_{HCW} + \rho_3\, I'_{HCW}$$

Table S1: Model variables

Variable description	Symbol	Notes
Susceptible hospitalised patients	S_H	Initial value: 999. (equal to one less than the total number of patients in the hospital, which is assumed not to vary)
Latently infected hospitalised patients	$E1_H$, $E2_H$	Initial value:0. Includes patients infected in the hospital and those infected in the community admitted for reasons other than COVID-19. Use of two compartments corresponds to an assumption of an Erlang-distributed latent period.
Infected and infectious	$I1_H$, $I2_H$	Initial value: 0.

hospitalised patients		Combines infectious patients who have become infected while in hospital and infectious patients admitted from the community for reasons other than COVID-19. Use of two compartments corresponds to an assumption of an Erlang-distributed infectious period.
Infected and infectious hospitalised patients with severe infections	I'_H	Initial value: 1. These patients are all assumed to be known to have COVID-19 and to be appropriately isolated.
Immune hospitalised patients	R_H	Initial value: 0. We assume only infection-derived sterilising immunity
Susceptible hospital healthcare workers (HCWs)	S_{HCW}	Initial value: 4000. (corresponding to the entire population of HCWs which is assumed not to vary)

Latently infected HCWs	$E1_{HCW}$, $E2_{HCW}$	Initial value: 0.
Infected and infectious HCWs who have not yet isolated	$I1_{HCW}$, $I2_{HCW}$	Initial value:0.
Infected HCWs in isolation	I'_{HCW}	Initial value: 0.<br>Infected patients who are isolated are assumed to no longer be able to infect hospitalised patients and other HCWs
Immune HCWs	R_{HCW}	Initial value: 0.
Susceptible people in the community	S_C	499,990.<br>(corresponding to 10 fewer than the entire number of people in the community which is assumed not to vary)
Latently infected people in the community	$E1_C$, $E2_C$	Initial value: 0.
Infected and infectious people in the community	$I1_C$, $I2_C$	Initial values<br>$I1_C$: 10.<br>$I2_C$: 0.
Immune people in the community	R_C	Initial value: 0.

Table S2: Model Parameters

Parameter description	Symbol	Values considered	Notes
Patient-to-patient single admission reproduction number. Expected number of secondary cases amongst hospitalised patients directly infected by one hospitalised infected unisolated patient in the absence of immunity amongst hospitalised patients	$R_{H\text{->}H}$	High hospital transmission scenario: 1.0<br><br>Intermediate hospital transmission scenario: 0.75<br><br>Low hospital transmission scenario: 0.50	Direct estimates are not available as only a proportion of hospital-acquired infections are observed, however regression coefficients from negative binomial autoregressive models (Table S3- S14) when interpreted in light of the simulation study (Extended data Figure 5) and estimated probabilities of detecting hospital acquired infection (Figure 1 D-F) are consistent with intermediate-to-high scenario values. A large genomic study

			at Sheffield Teaching Hospitals NHS Foundation Trust also gives estimates consistent with these scenarios[21], reporting a crude number of secondary cases per case of 0.40 (95% CI 0.31–0.48) for Wave 2 (30th November 2020 to 24th January 2021); after accounting for the detection probability of approximately 0.3 associated with the screening protocol (Figure 1D) and the approximately ten-fold higher number of secondary cases arising from patients with hospital-acquired

			infections compared to patients with community-acquired infections, these numbers are also in line with the high transmission scenario.
HCW-to-patient reproduction number. Expected number of secondary cases amongst hospitalised patients directly infected by one infected HCW in the absence of immunity amongst hospitalised patients	$R_{HCW\text{->}H}$	High hospital transmission scenario: 0.2<br><br>Intermediate hospital transmission scenario: 0.15<br><br>Low hospital transmission scenario: 0.1	Substantially lower onward transmission to patients from infected HCWs compared to nosocomially infected patients is consistent with both analysis of ward-level data [9], data from this study (Table S3- S14) and genomic studies [21].
Patient-to-HCW	$R_{H\text{->}HCW}$	High hospital	Parameter values

single admission reproduction number. Expected number of secondary cases amongst HCWs directly infected by one infected unisolated patient in the absence of immunity amongst HCWs patients during a single hospital admission		transmission scenario: 0.8<br><br>Intermediate hospital transmission scenario: 0.6<br><br>Low hospital transmission scenario: 0.4	chosen to be consistent with estimates from regression models in this paper (Tables S12-S14).
Community-to-community reproduction number. Expected number of secondary cases in the community directly infected by one infected person in the community in the absence of immunity	$R_{C\text{->}C}$	Before first lockdown: 3<br><br>After first lockdown: 0.6-1.2	Parameter value chosen to be illustrative of community transmission in an unmitigated epidemic and consistent with an estimated basic reproduction number early in the pandemic of 3.1 (95% CI 2.4,

			4.1) [40].
HCW-to-community reproduction number. Expected number of secondary cases in the community directly infected by one HCW in the absence of immunity	$R_{HCW->C}$	0.2	
HCW-to-HCW reproduction number. Expected number of secondary cases in HCWs directly infected by one HCW in the absence of immunity	$R_{HCW->HCW}$	High hospital transmission scenario: 0.5<br><br>Intermediate hospital transmission scenario: 0.375<br><br>Low hospital transmission scenario: 0.25	The assumption that each infected HCW generates about 60% of the secondary cases in HCWs as a nosocomially infected patient is consistent with analysis of ward-level data [9].
Community-to-HCW	$R_{C->HCW}$	0.1	Assumption consistent

reproduction number. Expected number of secondary cases in HCWs directly infected by one infected person in the community in the absence of immunity			with approximately 10-fold less transmission from patients with community acquired infection compared to patients with hospital-acquired infection, as estimated using genomic data [21].
Patient-to-patient transmission parameter	$\boldsymbol{\beta}_{\mathrm{H,H}}$	$R_{H\text{->}H}/D_H$	D_H is the mean duration of infectiousness while continuously hospitalised for a patient infected while in hospital and without severe infection. This is given by $(\gamma_1/(\gamma_1+\boldsymbol{\mu}))(\gamma_2/(\gamma_2+\boldsymbol{\mu}))$ x $(1/(\boldsymbol{\mu}+\rho_1)(1+\rho_1/(\boldsymbol{\mu}+\rho_2)$).

Patient-to-patient transmission parameter for patients hospitalised due to COVID-19	$\boldsymbol{\beta}_{H',H}$	0	We assume patients known to have COVID-19 are effectively isolated from non-COVID patients and not able to transmit to them.
Patient-to-HCW transmission parameter	$\boldsymbol{\beta}_{H,HCW}$	$R_{H\text{->}HCW}/D_H$	See above entry in this table for patient-to-patient transmission parameter for definition of D_H .
HCW-to-patient transmission parameter	$\boldsymbol{\beta}_{HCW,H}$	$R_{HCW\text{->}H}/D_{HCW}$	D_{HCW} is the mean duration an infected HCW remains infectious to the hospital population.
HCW-to-community transmission parameter	$\boldsymbol{\beta}_{HCW,C}$	$R_{HCW\text{->}C}/D_C$	D_C is the mean duration of infectiousness for someone in the community.
Community-to-HCW	$\boldsymbol{\beta}_{C,HCW}$	$R_{C\text{->}HCW}/D_C$	See above for the

transmission parameter			definition of D_C .
Community-to-community transmission parameter	$\boldsymbol{\beta}_{C,C}$	$R_{C\text{->}C}/D_C$	See above for the definition of D_C .
Hospital discharge rate for patients not hospitalised due to COVID-19	$\boldsymbol{\mu}$	0.2 /day	Mean length of stay is given by $1/\boldsymbol{\mu}$.
Hospital discharge rate for patients hospitalised due to COVID-19	$\boldsymbol{\mu}'$	0.05 /day	
Rate of admission to hospital for reasons other than COVID-19 for patients infected with SARS-CoV-2	$\boldsymbol{\alpha}$	0.001 /day	
Rate of admission to hospital for patients	$\boldsymbol{\alpha}'$	0.5 /day	

with COVID-19 infection requiring hospital treatment			
Progression rate from E1 states to E2 states	γ_1	0.5	Corresponds to a latent period with an Erlang distribution with a mean of four days.
Progression rate from E2 states to I1 states	γ_2	0.5	
Progression rate from I1 states to I2 states	ρ_1	0.3	Corresponds to an infectious period with an Erlang distribution with a mean of 6.67 days.
Progression rate from I2 states to R states	ρ_2	0.3	
Progression rate from I' states to R states	ρ_3	0.1	
Proportion of non-severe hospitalised infected patients who progress to severe infection	π_H	0.3	
Proportion of infected	π_C	0.1	

people in the community who progress to severe infection			
Proportion of infected HCWs who progress to severe infection	π_{HCW}	0.1	

1.3 Seroprevalence in healthcare-workers versus the community: international comparisons

To provide international comparisons, PubMed, Google Scholar and medRxiv were searched for literature published before 16 May 2021 with the main theme on seroprevalence in HCWs during the COVID-19 pandemic. The search terms used were: seroprevalence/antibody, healthcare worker, and SARS-CoV-2/COVID-19. Title, abstract, and full texts of the search results were reviewed. Estimated seroprevalence in HCWs was extracted from the papers and plotted against seroprevalence in the community. The seroprevalence in the community was extracted from the papers directly whenever data were available. For papers not reporting seroprevalence in the community over the same period we searched the literature for the estimates of seroprevalence in the community in the same country and over the most closely matching time period.

2 Supplementary Results

2.1 Quantifying the number of hospital-acquired infections: sensitivity analyses.

In the main analysis we used aggregate length-of-stay data from all acute trusts combined over a 12 month period. We performed four sensitivity analyses: i) using time-varying length-of-stay data (aggregated over 3 month intervals) and summing interval-specific estimates of hospital-acquired infections; ii) using trust-specific length-of-stay data in the estimation of the number of hospital-acquired infections in each trust; iii) using length-of-stay data that did not exclude patients with indeterminate healthcare-associated infection; and iv) using PCR sensitivity data estimated by Kucirka et al [41].

The use of time-varying length of stay data gave estimates (90% CrI) of 144,000 (125,000 , 170,000) hospital-acquired infections under the assumption of day 3 and day 6 screening, and 100,00 (96,000 , 105,00) under the assumption of weekly screening. Corresponding estimates with trust-specific length-of-stay data were 142,000 (123,000, 168,000) and 99,000 (96,000, 104,000). Trust-specific estimates excluded data from 8 trusts for which length-of-stay data were not available. These excluded trusts had a combined total of 180 "definite" healthcare-associated infections. The use of length-of-stay data that did not exclude patients with indeterminate healthcare-associated infection gave corresponding estimates of 142,000 (123,000, 166,000) and 98,000 (94,000, 103,000). Sensitivity analysis using PCR sensitivity estimates by Kucirka *et al* gave estimates of 143,000 (124,000, 167,000) and 99,000 (95,000, 104,000) [41].

In the main analysis we used as input the number of "definite" healthcare associated infections (those with onset 15 or more days after hospital admission) as input data and estimated the number of hospital-acquired infections by multiplying this by the reciprocal of the probability of hospital-

acquired infection being both detected and meeting the 15-day criterion (making use of the length-of-stay distribution, the incubation period distribution, the PCR sensitivity profile and the assumed testing policy in place, extending a previously-described analytical approach) [15]. As an additional sensitivity analysis we repeated this analysis using instead, as input data, the number of "probable and definite" healthcare associated infections (those with onset 8 or more days after hospital admission) and multiplied this by the inverse of the probability of a hospital-acquired infection being detected and meeting this criterion. With a policy of testing on symptom onset and at 7 day intervals after admission the estimated number of hospital acquired infections (90% CrI) was 122,000 (120,000 , 123,000) while with a policy of testing symptomatic infections and on days 3 and 6 post-admission the corresponding estimate was 189,000 (183,000, 196,000).

2.2 Full results from analysis with negative binomial auto-regressive models

Outcome 1: Indeterminate, probable and definite healthcare associated infections.

Table S3: Model P1.1.1

	mean	se_mean	sd	2.5%	25%	50%	75%	97.5%	n_eff	Rhat
a0 (intercept)	0.07	0.00	0.07	0.00	0.02	0.05	0.10	0.27	18514	1
d (community-acquired)	0.02	0.00	0.01	0.01	0.02	0.02	0.03	0.04	6880	1
b (hospital-acquired)	0.33	0.00	0.03	0.28	0.31	0.33	0.35	0.39	4795	1
c (HCW)	0.03	0.00	0.01	0.02	0.03	0.03	0.03	0.04	6251	1
q (single rooms)	-0.09	0.00	0.04	-0.17	-0.12	-0.09	-0.07	-0.02	5439	1
u (heated volume)	-0.13	0.00	0.04	-0.22	-0.16	-0.13	-0.10	-0.05	5610	1
s (occupancy)	0.00	0.00	0.03	-0.06	-0.02	0.00	0.02	0.06	5807	1
r (trust size)	0.12	0.00	0.02	0.07	0.10	0.11	0.13	0.16	5287	1

t (trust age)	-0.05	0.00	0.03	-0.10	-0.07	-0.05	-0.03	0.01	5352	1
v (vaccine)	-2.25	0.01	0.63	-3.50	-2.67	-2.25	-1.83	-1.05	13932	1
nv0	0.48	0.00	0.06	0.37	0.44	0.47	0.51	0.59	7239	1
sigmasq_a	5.94	0.01	0.94	4.18	5.30	5.91	6.55	7.88	4202	1
sigmasq_k	0.09	0.00	0.03	0.02	0.07	0.09	0.12	0.16	2515	1
phi0	0.27	0.00	0.03	0.21	0.24	0.26	0.29	0.34	10249	1
k0	0.10	0.00	0.02	0.06	0.09	0.10	0.11	0.15	2473	1

Leave-one-out information criterion: 10587.3

Table S4: Model P1.1.0

	mean	se_mean	sd	2.5%	25%	50%	75%	97.5%	n_eff	Rhat
a0 (intercept)	0.09	0.00	0.10	0.00	0.03	0.06	0.13	0.36	8515	1
d (community-acquired)	0.06	0.00	0.01	0.05	0.06	0.06	0.07	0.08	12021	1
b (hospital-acquired)	0.54	0.00	0.02	0.49	0.52	0.54	0.55	0.58	10291	1
c (HCW)	0.03	0.00	0.01	0.02	0.03	0.03	0.04	0.04	4709	1
q (single rooms)	-0.11	0.00	0.03	-0.16	-0.13	-0.11	-0.09	-0.06	12190	1
u (heated volume)	-0.14	0.00	0.03	-0.20	-0.16	-0.14	-0.12	-0.08	11944	1
s (occupancy)	0.00	0.00	0.02	-0.04	-0.01	0.00	0.02	0.04	12423	1
r (trust size)	0.06	0.00	0.02	0.02	0.04	0.06	0.07	0.09	5720	1
t (trust age)	-0.06	0.00	0.02	-0.10	-0.07	-0.06	-0.04	-0.02	15728	1
sigmasq_a	1.19	0.01	0.70	0.08	0.65	1.13	1.64	2.72	2495	1
sigmasq_k	0.09	0.00	0.03	0.03	0.07	0.09	0.10	0.14	3249	1
phi0	0.39	0.00	0.05	0.30	0.36	0.38	0.42	0.48	13168	1
k0	0.07	0.00	0.02	0.04	0.06	0.07	0.08	0.11	3069	1

Leave-one-out information criterion:10591.7

Table S5: Model P1.0.0

	mean	se_mean	sd	2.5%	25%	50%	75%	97.5%	n_eff	Rhat
a0 (intercept)	0.06	0.00	0.07	0.00	0.02	0.04	0.08	0.25	7155	1
d (community-acquired)	0.07	0.00	0.01	0.05	0.06	0.07	0.08	0.09	12690	1
b (hospital-acquired)	0.60	0.00	0.02	0.55	0.58	0.60	0.61	0.64	9730	1
c (HCW)	0.03	0.00	0.01	0.02	0.02	0.03	0.03	0.04	3679	1
sigmasq_a	1.29	0.02	0.75	0.08	0.72	1.26	1.80	2.86	1896	1
sigmasq_k	0.09	0.00	0.03	0.04	0.07	0.09	0.11	0.14	2752	1
phi0	0.38	0.00	0.04	0.30	0.35	0.37	0.40	0.47	12115	1
k0	0.07	0.00	0.02	0.04	0.06	0.07	0.08	0.10	2484	1

Leave-one-out information criterion: 10607.6

Outcome 2: Probable and definite healthcare associated infections.

Probable and definite healthcare associated infections are those with onset/detection eight or more days after the day of hospital admission.

Table S6: Model P1.1.1

	mean	se_mean	sd	2.5%	25%	50%	75%	97.5%	n_eff	Rhat
a0 (intercept)	0.08	0.00	0.08	0.00	0.02	0.06	0.11	0.30	13074	1
d (community-acquired)	0.02	0.00	0.01	0.01	0.02	0.02	0.03	0.03	5645	1
b (hospital-acquired)	1.07	0.00	0.07	0.93	1.02	1.07	1.11	1.19	5523	1
c (HCW)	0.03	0.00	0.00	0.02	0.02	0.03	0.03	0.04	4199	1
q (single rooms)	-0.09	0.00	0.03	-0.14	-0.11	-0.09	-0.07	-0.03	10190	1
u (heated volume)	-0.10	0.00	0.03	-0.17	-0.12	-0.10	-0.07	-0.03	9945	1
s (occupancy)	0.01	0.00	0.02	-0.04	-0.01	0.01	0.02	0.05	13533	1
r (trust size)	0.04	0.00	0.02	0.01	0.03	0.04	0.05	0.08	3758	1
t (trust age)	-0.04	0.00	0.02	-0.08	-0.05	-0.04	-0.03	0.00	13427	1

v (vaccine)	-2.00	0.01	0.59	-3.16	-2.40	-1.99	-1.59	-0.85	11032	1
w (alpha variant)	0.18	0.00	0.06	0.07	0.14	0.18	0.22	0.30	5604	1
sigmasq_a	0.40	0.01	0.44	0.01	0.10	0.26	0.56	1.64	1621	1
sigmasq_k	0.12	0.00	0.04	0.03	0.09	0.12	0.14	0.20	1993	1
phi0	0.28	0.00	0.04	0.22	0.26	0.28	0.31	0.36	9354	1
k0	0.11	0.00	0.03	0.06	0.09	0.11	0.13	0.17	1909	1

Leave-one-out information criterion: 8884.7

Table S7: Model P1.1.0

	mean	se_mean	sd	2.5%	25%	50%	75%	97.5%	n_eff	Rhat
a0 (intercept)	0.08	0	0.07	0.00	0.02	0.05	0.11	0.27	11513	1
d (community-acquired)	0.03	0	0.01	0.02	0.03	0.03	0.04	0.04	11151	1
b (hospital-acquired)	0.60	0	0.03	0.55	0.58	0.60	0.62	0.65	12953	1
c (HCW)	0.02	0	0.00	0.01	0.02	0.02	0.02	0.03	5790	1
q (single rooms)	-0.10	0	0.03	-0.16	-0.12	-0.10	-0.08	-0.04	13043	1
u (heated volume)	-0.10	0	0.03	-0.17	-0.13	-0.10	-0.08	-0.04	11074	1
s (occupancy)	0.01	0	0.02	-0.03	0.00	0.01	0.03	0.06	14400	1
r (trust size)	0.04	0	0.02	0.01	0.03	0.04	0.05	0.07	7975	1
t (trust age)	-0.04	0	0.02	-0.08	-0.06	-0.04	-0.03	0.00	14289	1
sigmasq_a	0.33	0	0.28	0.01	0.11	0.25	0.46	1.05	3674	1
sigmasq_k	0.11	0	0.04	0.03	0.09	0.12	0.14	0.19	2235	1
phi0	0.33	0	0.04	0.26	0.30	0.33	0.36	0.42	13389	1
k0	0.10	0	0.03	0.06	0.08	0.10	0.12	0.16	2074	1

Leave-one-out information criterion: 9196.4

Table S8: Model P1.0.0

	mean	se_mean	sd	2.5%	25%	50%	75%	97.5%	n_eff	Rhat
a0 (intercept)	0.06	0.00	0.06	0.00	0.02	0.04	0.08	0.23	10293	1
d (community-acquired)	0.04	0.00	0.01	0.02	0.03	0.04	0.04	0.05	15490	1
b (hospital-acquired)	0.66	0.00	0.03	0.61	0.64	0.66	0.68	0.71	15446	1
c (HCW)	0.02	0.00	0.00	0.01	0.02	0.02	0.02	0.03	5272	1
sigmasq_a	0.41	0.01	0.33	0.01	0.15	0.34	0.61	1.20	3448	1
sigmasq_k	0.12	0.00	0.04	0.03	0.09	0.12	0.14	0.19	2292	1
phi0	0.32	0.00	0.04	0.25	0.29	0.32	0.34	0.40	14926	1
k0	0.10	0.00	0.03	0.06	0.08	0.10	0.11	0.16	2020	1

Leave-one-out information criterion: 9207.9

Outcome 3: Definite healthcare associated infections.

Definite healthcare associated infections are those with onset/detection 15 or more days after the day of hospital admission.

Table S9: Model P1.1.1

	mean	se_mean	sd	2.5%	25%	50%	75%	97.5%	n_eff	Rhat
a0 (intercept)	0.04	0.00	0.04	0.00	0.01	0.03	0.06	0.15	13701	1
d (community-acquired)	0.01	0.00	0.00	0.01	0.01	0.01	0.02	0.02	7688	1
b (hospital-acquired)	0.56	0.00	0.04	0.49	0.54	0.56	0.58	0.63	8462	1
c (HCW)	0.01	0.00	0.00	0.01	0.01	0.01	0.02	0.02	4488	1
q (single rooms)	-0.10	0.00	0.03	-0.16	-0.12	-0.10	-0.07	-0.03	9970	1
u (heated volume)	-0.06	0.00	0.04	-0.14	-0.09	-0.06	-0.04	0.02	9598	1
s (occupancy)	0.02	0.00	0.03	-0.04	0.00	0.02	0.04	0.08	11123	1

r (trust size)	0.03	0.00	0.02	-0.01	0.01	0.03	0.04	0.07	6889	1
t (trust age)	-0.04	0.00	0.02	-0.09	-0.05	-0.04	-0.02	0.01	12478	1
v (vaccine)	-1.64	0.01	0.63	-2.89	-2.06	-1.63	-1.21	-0.43	10917	1
w (alpha variant)	0.08	0.00	0.07	-0.05	0.03	0.08	0.12	0.22	7235	1
sigmasq_a	0.13	0.00	0.13	0.00	0.03	0.09	0.18	0.47	3272	1
sigmasq_k	0.35	0.00	0.07	0.21	0.30	0.34	0.39	0.50	3992	1
phi0	0.34	0.00	0.04	0.26	0.31	0.33	0.36	0.43	10230	1
k0	0.07	0.00	0.03	0.02	0.05	0.07	0.09	0.15	2608	1

Leave-one-out information criterion: 7380.5

Table S10: Model P1.1.0

	mean	se_mean	sd	2.5%	25%	50%	75%	97.5%	n_eff	Rhat
a0 (intercept)	0.04	0	0.04	0.00	0.01	0.03	0.06	0.15	12237	1
d (community-acquired)	0.02	0	0.00	0.01	0.02	0.02	0.02	0.03	9555	1
b (hospital-acquired)	1.02	0	0.05	0.91	0.98	1.02	1.05	1.12	12117	1
c (HCW)	0.01	0	0.00	0.01	0.01	0.01	0.01	0.02	4661	1
q (single rooms)	-0.11	0	0.04	-0.18	-0.14	-0.11	-0.09	-0.05	11102	1
u (heated volume)	-0.08	0	0.04	-0.16	-0.11	-0.08	-0.05	0.00	10383	1
s (occupancy)	0.02	0	0.03	-0.03	0.00	0.02	0.04	0.08	12640	1
r (trust size)	0.04	0	0.02	0.00	0.02	0.04	0.05	0.08	7417	1
t (trust age)	-0.05	0	0.03	-0.10	-0.06	-0.05	-0.03	0.00	14738	1
sigmasq_a	0.16	0	0.16	0.00	0.04	0.11	0.22	0.57	2838	1
sigmasq_k	0.32	0	0.07	0.19	0.28	0.32	0.37	0.47	4855	1
phi0	0.37	0	0.04	0.29	0.34	0.37	0.40	0.46	13262	1
k0	0.07	0	0.03	0.02	0.05	0.07	0.09	0.14	3477	1

Leave-one-out information criterion: 7396.1

Table S11: Model P1.0.0

	mean	se_mean	sd	2.5%	25%	50%	75%	97.5%	n_eff	Rhat
a0 (intercept)	0.03	0	0.03	0.00	0.01	0.02	0.04	0.13	9101	1
d (community-acquired)	0.02	0	0.00	0.01	0.02	0.02	0.02	0.03	11485	1
b (hospital-acquired)	0.63	0	0.03	0.58	0.61	0.63	0.65	0.69	13361	1
c (HCW)	0.01	0	0.00	0.00	0.01	0.01	0.01	0.01	3871	1
sigmasq_a	0.29	0	0.18	0.01	0.14	0.27	0.40	0.67	3020	1
sigmasq_k	0.35	0	0.07	0.22	0.30	0.35	0.40	0.49	6169	1
phi0	0.35	0	0.04	0.28	0.32	0.35	0.38	0.45	9178	1
k0	0.06	0	0.03	0.01	0.04	0.06	0.08	0.13	3820	1

Leave-one-out information criterion: 7390.5

Outcome 4: HCW infections.

HCW infections are imputed from trust data on the number of HCWs absent due to COVID-19 on each day, assuming that each absence lasts for a period of 10 days.

Table S12: Model P1.1.1

	mean	se_mean	sd	2.5%	25%	50%	75%	97.5%	n_eff	Rhat
a0 (intercept)	9.36	0.01	0.95	7.47	8.71	9.36	10.01	11.24	9942	1
d (community-acquired)	0.08	0.00	0.01	0.06	0.07	0.08	0.09	0.11	22430	1
b (hospital-acquired)	0.89	0.00	0.11	0.67	0.81	0.89	0.96	1.11	24263	1
c (HCW)	0.07	0.00	0.01	0.05	0.06	0.07	0.07	0.08	22559	1
q (single rooms)	0.06	0.00	0.04	-0.01	0.04	0.06	0.09	0.13	7433	1
u (heated volume)	-0.01	0.00	0.04	-0.09	-0.03	0.00	0.03	0.08	7482	1
s (occupancy)	0.03	0.00	0.03	-0.04	0.00	0.03	0.05	0.10	7262	1
r (trust size)	0.39	0.00	0.03	0.34	0.37	0.39	0.41	0.45	6482	1
t (trust age)	0.01	0.00	0.03	-0.05	-0.01	0.01	0.03	0.07	7345	1

	mean	se_mean	sd	2.5%	25%	50%	75%	97.5%	n_eff	Rhat
v (vaccine)	-0.30	0.00	0.19	-0.67	-0.43	-0.30	-0.18	0.07	16531	1
w (alpha variant)	0.89	0.00	0.06	0.78	0.85	0.89	0.93	1.00	14827	1
sigmasq_a	18.45	0.00	0.65	17.16	18.01	18.45	18.89	19.71	17039	1
sigmasq_k	0.02	0.00	0.00	0.02	0.02	0.02	0.02	0.03	9670	1
phi0	0.99	0.00	0.08	0.84	0.94	0.99	1.04	1.14	11187	1
k0	0.00	0.00	0.00	0.00	0.00	0.00	0.00	0.00	26116	1

Leave-one-out information criterion: 17804.3

Table S13: Model P1.1.0

	mean	se_mean	sd	2.5%	25%	50%	75%	97.5%	n_eff	Rhat
a0 (intercept)	9.39	0.01	1.00	7.44	8.71	9.39	10.06	11.35	10288	1
d (community-acquired)	0.15	0.00	0.01	0.13	0.14	0.15	0.16	0.17	15782	1
b (hospital-acquired)	0.71	0.00	0.06	0.60	0.67	0.71	0.75	0.83	16005	1
c (HCW)	0.09	0.00	0.01	0.07	0.08	0.09	0.09	0.10	14377	1
q (single rooms)	0.03	0.00	0.03	-0.04	0.01	0.03	0.05	0.10	5070	1
u (heated volume)	-0.01	0.00	0.04	-0.10	-0.04	-0.01	0.02	0.07	5131	1
s (occupancy)	0.10	0.00	0.04	0.03	0.07	0.09	0.12	0.16	5319	1
r (trust size)	0.39	0.00	0.03	0.34	0.37	0.39	0.42	0.46	3864	1
t (trust age)	0.00	0.00	0.03	-0.06	-0.02	0.00	0.02	0.06	5867	1
sigmasq_a	20.84	0.01	0.66	19.57	20.40	20.84	21.28	22.13	12798	1
sigmasq_k	0.02	0.00	0.00	0.02	0.02	0.02	0.02	0.03	7808	1
phi0	0.93	0.00	0.07	0.80	0.88	0.93	0.97	1.06	10680	1
k0	0.00	0.00	0.00	0.00	0.00	0.00	0.00	0.00	25752	1

Leave-one-out information criterion: 17867.3

Table S14: Model P1.0.0

	mean	se_mean	sd	2.5%	25%	50%	75%	97.5%	n_eff	Rhat
a0 (intercept)	9.27	0.01	0.95	7.42	8.61	9.28	9.91	11.11	9802	1

d (community-acquired)	0.16	0.00	0.01	0.14	0.15	0.16	0.17	0.19	22771	1
b (hospital-acquired)	0.78	0.00	0.06	0.67	0.74	0.78	0.82	0.90	21310	1
c (HCW)	0.10	0.00	0.01	0.09	0.10	0.10	0.11	0.12	18849	1
sigmasq_a	20.79	0.01	0.69	19.43	20.32	20.79	21.25	22.14	16335	1
sigmasq_k	0.02	0.00	0.00	0.01	0.02	0.02	0.02	0.02	9573	1
phi0	0.94	0.00	0.07	0.81	0.90	0.94	0.99	1.08	10377	1
k0	0.00	0.00	0.00	0.00	0.00	0.00	0.00	0.00	25615	1

Leave-one-out information criterion: 18090.4

Outcome 5: Negative control outcome - admissions of patients with confirmed community-acquired Covid-19 infections.

Results below give output from auto-regression models where the dependent variable is a negative control given by the weekly number of confirmed community-acquired COVID-19 admissions to each trust. This is a negative control in the sense that many factors that might be supposed to causally affect the rate of hospital transmission in a trust (such as factors related to the hospital buildings) would not, in general, be expected to have a large impact on the admissions of community-acquired cases. For such covariates associations of similar magnitude and in the same direction for both the outcome of interest and for the negative control outcome can therefore provide evidence against there being a direct causal effect.

Table S15: Model P1.1.1

	mean	se_mean	sd	2.5%	25%	50%	75%	97.5%	n_eff	Rhat
adm_coeff	0.29	0.00	0.02	0.25	0.27	0.29	0.30	0.32	749	1
b (hospital-acquired)	0.04	0.00	0.02	0.01	0.03	0.04	0.06	0.08	779	1
c (HCW)	0.01	0.00	0.00	0.00	0.01	0.01	0.01	0.02	186	1
q (single rooms)	0.03	0.00	0.04	-0.05	0.00	0.03	0.05	0.09	266	1

u (heated volume)	-0.09	0.00	0.05	-0.18	-0.12	-0.09	-0.06	0.02	286	1
s (occupancy)	0.06	0.00	0.04	-0.02	0.03	0.06	0.09	0.14	182	1
r (trust size)	0.11	0.00	0.02	0.06	0.09	0.11	0.12	0.15	341	1
t (trust age)	0.02	0.00	0.03	-0.04	0.00	0.02	0.04	0.08	361	1
v (vaccine)	-1.42	0.03	0.73	-2.85	-1.92	-1.43	-0.91	-0.03	522	1
w (alpha variant)	0.86	0.00	0.05	0.77	0.83	0.85	0.89	0.95	721	1
sigmasq_k	0.54	0.00	0.09	0.37	0.48	0.54	0.60	0.72	518	1
phi0	0.11	0.00	0.02	0.07	0.09	0.11	0.12	0.16	463	1
k0	0.07	0.00	0.03	0.02	0.05	0.06	0.08	0.12	237	1

Leave-one-out information criterion: 7838.9

Table S16: Model P1.1.0

	mean	se_mean	sd	2.5%	25%	50%	75%	97.5%	n_eff	Rhat
adm_coeff	0.41	0	0.03	0.36	0.39	0.41	0.43	0.46	342	1.01
b (hospital-acquired)	0.15	0	0.03	0.09	0.13	0.15	0.17	0.21	441	1.00
c (HCW)	0.02	0	0.00	0.01	0.01	0.02	0.02	0.02	302	1.00
q (single rooms)	-0.10	0	0.04	-0.19	-0.13	-0.10	-0.08	-0.02	223	1.00
u (heated volume)	-0.05	0	0.06	-0.17	-0.09	-0.05	-0.01	0.06	207	1.00
s(occupancy)	0.17	0	0.04	0.08	0.14	0.17	0.19	0.25	175	1.00
r (trust size)	0.12	0	0.02	0.08	0.11	0.12	0.14	0.17	331	1.00
t (trust age)	0.02	0	0.04	-0.07	0.00	0.02	0.05	0.10	166	1.00
sigmasq_k	0.41	0	0.06	0.31	0.37	0.41	0.45	0.54	363	1.00
phi0	0.11	0	0.02	0.07	0.09	0.10	0.12	0.14	723	1.00
k0	0.03	0	0.01	0.01	0.02	0.03	0.04	0.06	233	1.02

Leave-one-out information criterion: 8165.4

Table S17: Model P1.0.0

	mean	se_mean	sd	2.5%	25%	50%	75%	97.5%	n_eff	Rhat
a0 (intercept)	0.11	0.00	0.11	0.00	0.03	0.08	0.15	0.42	1568	1
adm_coeff	0.42	0.00	0.03	0.37	0.41	0.42	0.44	0.48	955	1
b (hospital-acquired)	0.19	0.00	0.03	0.13	0.17	0.19	0.21	0.25	1198	1
c (HCW)	0.02	0.00	0.00	0.01	0.02	0.02	0.03	0.03	719	1
sigmasq_a	13.60	0.09	1.91	10.39	12.22	13.43	14.88	17.55	485	1
sigmasq_k	0.44	0.00	0.06	0.33	0.40	0.43	0.47	0.56	617	1
phi0	0.10	0.00	0.02	0.07	0.09	0.10	0.11	0.14	1536	1
k0	0.01	0.00	0.01	0.00	0.01	0.01	0.02	0.03	237	1

Leave-one-out information criterion: 8194.5

Sensitivity analysis with outcome 2 (probable and definite healthcare associated infections) and a degree 3 spline model allowing for time-varying changes in the number of hospital-acquired infections not accounted for by the covariates.

Table S18 Model P1.1.1.tv

	Mean	se_mean	sd	2.5%	25%	50%	75%	97.5%	n_eff	Rhat
a0 (intercept)	0.02	0.00	0.02	0.00	0.01	0.02	0.03	0.08	8971	1
d (community-acquired)	0.01	0.00	0.00	0.00	0.01	0.01	0.01	0.02	5696	1
b (hospital-acquired)	0.42	0.00	0.08	0.28	0.36	0.42	0.47	0.59	3658	1
c (HCW)	0.01	0.00	0.00	0.00	0.01	0.01	0.01	0.01	3885	1
q (single rooms)	-0.07	0.00	0.03	-0.12	-0.09	-0.07	-0.05	-0.01	10711	1
s (occupancy)	0.00	0.00	0.02	-0.04	-0.01	0.00	0.02	0.05	13079	1
r (trust size)	0.03	0.00	0.02	-0.01	0.015	0.03	0.04	0.06	5120	1
t (trust age)	-0.03	0.00	0.02	-0.07	-0.05	-0.03	-0.02	0.01	13979	1
u (heated volume)	-0.08	0.00	0.03	-0.15	-0.11	-0.08	-0.06	-0.02	11399	1
v (vaccine)	0.22	0.00	0.72	-1.29	-0.24	0.24	0.71	1.56	12849	1
w (alpha variant)	0.21	0.00	0.12	-0.02	0.13	0.21	0.30	0.45	8135	1
phi0	0.34	0.00	0.04	0.26	0.31	0.34	0.37	0.44	10277	1
k0	0.11	0.00	0.03	0.06	0.09	0.11	0.13	0.18	1548	1

Leave-one-out information criterion: 8968.8

The estimated spline function is shown in Figure S1 below.

Note that the leave-one-out information criterion strongly favours the corresponding model but without the spline component (P1.1.1) which has a leave-one-out information criterion of 8884.7.

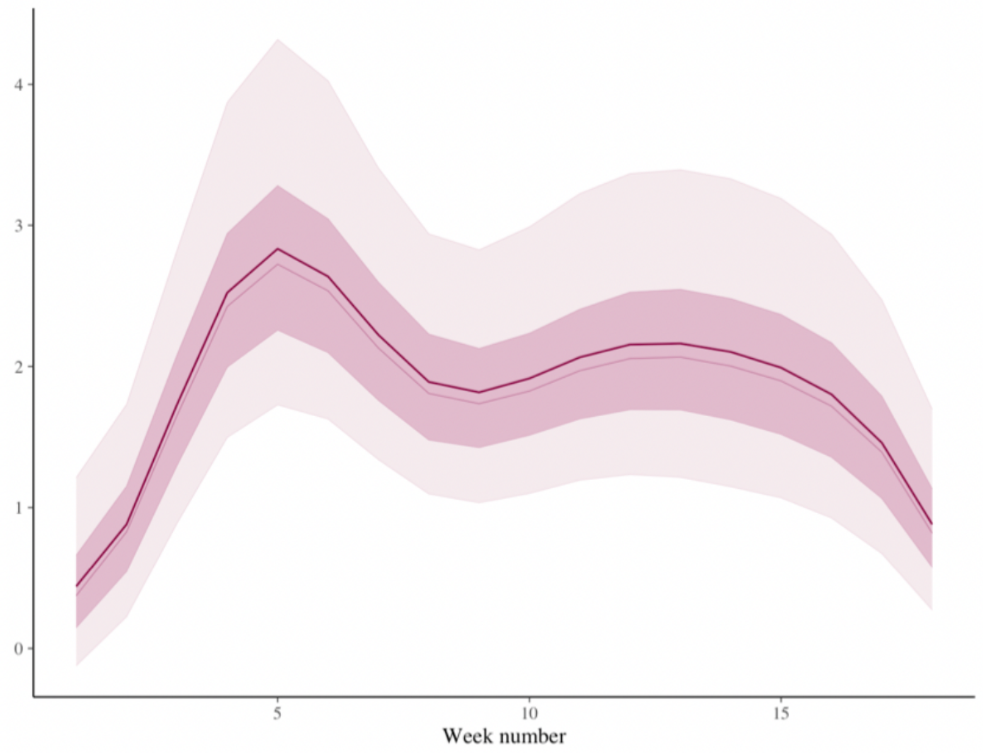

Figure S1 **| Estimated spline function from Model P1.1.1.tv.**

Here the dependent variable is probable and definite healthcare associated infection. Shaded regions correspond to 50% and 90% credible intervals. The spline has degree 3 and 6 equally-spaced knots. Note that the simpler model without the spline function (Model P1.1.1) has a substantially lower leave-one-out information criterion (8884.7 versus 8968.8).

2.3 Generation and analysis of synthetic data

Synthetic data were generated using three steps:

1. Fully observed daily infection data were generated for the number of infected HCWs and the number of hospital-acquired infections. This was done by, for each infected individual

of type x (where x may be a patient infected in the community (C), a patient infected in hospital (P), or a HCW (H)) : i) generating a number of secondary cases sampled from a negative binomial distribution with mean $R_{x,H}$+$R_{x,P}$ and dispersion parameter 0.5, where $R_{x,P}$and $R_{x,H}$represent the expected number of secondary cases in patients and, respectively, HCWs, generated by one infected individual of type x; ii) assigning these new infections to patients and HCWs by sampling from binomial distributions where the number of trials was equal to the number of secondary cases generated in i) and the probability the infection was in a patient was given by $R_{x,P}/(R_{x,H} + R_{x,P})$; iii) choosing the day of new infections by sampling generation intervals from a Weibull distribution with shape 2.83 and scale 5.67 [42].

2. Sampling the number of observed infections of each type for each day from binomial distributions where the number of trials was equal to the number of infections of each type and each day (generated in step 1) and the probability of "success" was the probability of observing (and correctly classifying) each type of infection (which was varied in the simulations).
3. Weekly aggregated data for each trust were created by summing the number of observed infections generated in step 2 over seven day intervals within each trust.

The above procedure was used to generate synthetic data for each trust, taking as input the daily number of observed community-acquired infections for each trust, and generating as output the weekly number of observed hospital-acquired infections in patients and HCWs. Simulated data thus generated were then analysed with negative binomial regression models with an identity link function where the dependent variable was either the simulated number of observed hospital-acquired infections or the simulated number of infections in HCWs observed for trust i in week j and where the independent variables were the numbers of observed HCW infections, hospital-

acquired patient infections and admitted patients with community-acquired infections in trust i and week $j-1$.

2.4 Vaccine coverage in patients

The regression analysis is restricted to the period between week 42 (beginning 14th October 2020) and week 55 (beginning 13th January 2021). In England, vaccine rollout to the over 70s and clinically extremely vulnerable began on 18 January 2021, while residents in care homes for older adults and their carers and all those aged 80 and over were first eligible for vaccination on 8th December 2020. If we assume that those vaccinated with the first dose two or more weeks previously have some degree of immunity, we can consider anyone vaccinated by the end of December 2020 to be partially vaccine-protected by the start of week 55. The NHS reported the total number of people in England who had been vaccinated by 27th December to be 786,000, and these vaccinations occurred in the following priority groups: care home residents and their carers, frontline health and social care workers and people aged 80 years old and over. 524,439 of those vaccinated by this time point are reported to be aged 80 and over. In 2020 there were 2.9 million people aged 80 years or over living in England, so 18% of those aged 80 and over can be assumed to have had some degree of vaccine protection by week 55. Of the 261,561 people who were vaccinated by 27th December and who were not aged 80 or over, we do not have a breakdown of the number who were care home residents, carers etc but at this time the number of people in England aged 70-79 was 4.8 million so at most 10% (i.e. 786000/ (4800000+2900000)) of those aged 70 and over in England had been vaccinated with at least one dose two or more weeks previously by week 55.

Data sources:

https://www.england.nhs.uk/statistics/wp-content/uploads/sites/2/2020/12/COVID-19-total-announced-vaccinations-31-December-2020.pdf

https://www.statista.com/statistics/281208/population-of-the-england-by-age-group/

2.5 Seroprevalence in HCWs versus the community: international comparisons

A total of 195 relevant studies and 1 systematic review paper were found. Of the 195 studies, 110 were done in Europe (of which 23 were done in the United Kingdom), 40 papers reported seroprevalence among HCWs in North America, 31 studies were in Asian countries, 8 studies were in African countries, 2 studies were in Turkey, 1 study was from Russia, 1 study was from South America, 1 study was from Australia, and 1 was a multinational study (Extended data Fig. 8). The studies varied in sample size from 24 to 61,910 HCWs. The type of antibody detected in the seroprevalence studies included IgA, IgM and IgG, and some used in-house antibiotic tests, while others used commercial point of care tests. Overall, the seroprevalence in HCWs ranged from 0% to 81%, and that in the general community ranged from 0.1% to 73%.

References

40. Read, J. M., Bridgen, J. R. E., Cummings, D. A. T., Ho, A. & Jewell, C. P. Novel coronavirus 2019-nCoV (COVID-19): early estimation of epidemiological parameters and epidemic size estimates. Philos. Trans. R. Soc. Lond. B Biol. Sci. 376, 20200265 (2021).

41. Kucirka, L. M., Lauer, S. A., Laeyendecker, O., Boon, D. & Lessler, J. Variation in false-negative rate of reverse transcriptase polymerase chain reaction-based SARS-CoV-2 tests by time since exposure. Ann. Intern. Med. 173, 262–267 (2020).

42. Ferretti, L. et al. Quantifying SARS-CoV-2 transmission suggests epidemic control with digital contact tracing. Science 368, eabb6936 (2020).
