# Supplementary material for: The burden and dynamics of hospital-acquired SARS-CoV-2 in England
Source: Nature. 2023 Oct 18;623(7985):132–8. doi: 10.1038/s41586-023-06634-z (PMC10620085; doi:10.1038/s41586-023-06634-z)
Supplement: Supplementary file 2 — Reporting Summary [file 41586_2023_6634_MOESM2_ESM.pdf]

nature portfolio

Corresponding author(s): Ben Cooper

Last updated by author(s): Jul 8, 2023

Reporting Summary

Nature Portfolio wishes to improve the reproducibility of the work that we publish. This form provides structure for consistency and transparency in reporting. For further information on Nature Portfolio policies, see our Editorial Policies and the Editorial Policy Checklist.

Statistics

For all statistical analyses, confirm that the following items are present in the figure legend, table legend, main text, or Methods section.

n/a	Confirmed	
☐	☒	The exact sample size (*n*) for each experimental group/condition, given as a discrete number and unit of measurement
☐	☒	A statement on whether measurements were taken from distinct samples or whether the same sample was measured repeatedly
☒	☐	The statistical test(s) used AND whether they are one- or two-sided<br>*Only common tests should be described solely by name; describe more complex techniques in the Methods section.*
☐	☒	A description of all covariates tested
☐	☒	A description of any assumptions or corrections, such as tests of normality and adjustment for multiple comparisons
☐	☒	A full description of the statistical parameters including central tendency (e.g. means) or other basic estimates (e.g. regression coefficient) AND variation (e.g. standard deviation) or associated estimates of uncertainty (e.g. confidence intervals)
☒	☐	For null hypothesis testing, the test statistic (e.g. *F*, *t*, *r*) with confidence intervals, effect sizes, degrees of freedom and *P* value noted<br>*Give P values as exact values whenever suitable.*
☐	☒	For Bayesian analysis, information on the choice of priors and Markov chain Monte Carlo settings
☐	☒	For hierarchical and complex designs, identification of the appropriate level for tests and full reporting of outcomes
☐	☒	Estimates of effect sizes (e.g. Cohen's *d*, Pearson's *r*), indicating how they were calculated

Our web collection on statistics for biologists contains articles on many of the points above.

Software and code

Policy information about availability of computer code

Data collection	All data used in this study were either derived from publicly available data sources or shared with us privately through the Scientific Pandemic Influenza Group on Modelling Operational sub-group (SPI-M-O) and information on software used to collect data was not provided.
Data analysis	All analysis was performed using R version 4.2.0 using the rstan package version 2.21.1 for the regression models. Code written for this analysis is available at the following URL: https://zenodo.org/record/8123987. Code to reconstruct the PCR sensitivity profile is available from https://github.com/cmmid/pcr-profile.

For manuscripts utilizing custom algorithms or software that are central to the research but not yet described in published literature, software must be made available to editors and reviewers. We strongly encourage code deposition in a community repository (e.g. GitHub). See the Nature Portfolio guidelines for submitting code & software for further information.

Data

Policy information about availability of data

All manuscripts must include a data availability statement. This statement should provide the following information, where applicable:

- Accession codes, unique identifiers, or web links for publicly available datasets
- A description of any restrictions on data availability
- For clinical datasets or third party data, please ensure that the statement adheres to our policy

The data that support the findings of this study are available as described below. Infection data used for this analysis were taken from daily situation reports

between 10th June 2020 and 17th February 2021 and shared privately with the Scientific Pandemic Influenza Group on Modelling Operational sub-group (SPI-M-O). The start date was chosen as the first date that healthcare-associated infections were consistently reported across trusts, and the end date was taken to be one month after the start of vaccine roll-out to the over 70s and clinically extremely vulnerable (18th January 2021). COVID-19 admission data for NHS trusts are publicly available by direct download from https://www.england.nhs.uk/statistics/statistical-work-areas/covid-19-hospital-activity/. Requests for data on healthcare associated infections should be sent to Dr Julie Robotham (julie.robotham@phe.gov.uk) who will liaise with NHS England to provide access to these data and will respond to requests within one month. Trust-specific data used in the analysis not related to infections (number of single rooms, size, age, heated volume and bed occupancy) were derived from the Estates Returns Information Collection from NHS Digital (available for download at https://digital.nhs.uk/data-and-information/publications/statistical/estates-returns-information-collection) including only the following site types: general acute hospital, community hospital (with inpatient beds), mixed service hospital, specialist hospital (acute only). The number of single rooms was expressed as the number of beds in single rooms in the trust (including single bedrooms for patients with and without en-suite facilities and isolation rooms) divided by the number of general and acute beds reported as being available in the trust in the last quarter of 2020. Hospital size was taken as the number of hospital beds available in the trust. A hospital building age score was taken as a weighted average of the proportion of floor area across hospital sites that was built before 1965, where weights were taken as the building floor area.
Data relating to vaccine coverage in healthcare workers were collected as part of the SIREN study (ISRCTN Number. ISRCTN11041050). Data from this study are available on reasonable request to Dr Julie Robotham and will be available through the Health Data Research UK CO-CONNECT platform and available for secondary analysis once the SIREN study has completed reporting. Using these data we classified healthcare workers as being immunised if they had received at least one vaccine dose three or more weeks previously. Otherwise they were considered un-immunised. SARS-CoV-2 variant data consisted of the proportion of characterised isolates that were attributed to the Alpha variant in each week for each NHS region. The prevalence of the Alpha variant by region and over time was determined by the proportion of tests with S-gene target failure status from PCR tests provided by Public Health England accessed at (https://github.com/epiforecasts/covid19.sgene.utla.rt)36. Patient length of stay data were taken from Secondary Uses Service (SUS)37. Data to reconstruct the PCR sensitivity profile are available from https://github.com/cmmid/pcr-profile.

Human research participants

Policy information about studies involving human research participants and Sex and Gender in Research.

Reporting on sex and gender	Neither sex nor gender were considered in the analysis and we did not have access to data relating to sex or gender of those infected or at risk of infection.
Population characteristics	We did not consider age or other individual characteristics of patients
Recruitment	The study was a retrospective analysis of national datasets for England, so patients were not recruited into this study.
Ethics oversight	The study did not involve the collection of new patient data, or use any personal identifiable information, but used a combination of anonymised national aggregate data sources including C19SR01 - COVID-19 Daily NHS Provider SitRep, and regionally aggregated vaccine coverage data from the SIREN study for which the study protocol was approved by the Berkshire Research Ethics Committee on May 22, 2020 with the vaccine amendment approved on Dec 23, 2020.

Note that full information on the approval of the study protocol must also be provided in the manuscript.

Field-specific reporting

Please select the one below that is the best fit for your research. If you are not sure, read the appropriate sections before making your selection.

☒ Life sciences ☐ Behavioural & social sciences ☐ Ecological, evolutionary & environmental sciences

For a reference copy of the document with all sections, see nature.com/documents/nr-reporting-summary-flat.pdf

Life sciences study design

All studies must disclose on these points even when the disclosure is negative.

Sample size	This was a retrospective analysis of complete national data rather than a sample, and all adult NHS hospital Trusts in England with available data were included. This represented over 98% of the total NHS general and acute care bed capacity in England in 2020. .
Data exclusions	Date ranges were chosen to start when healthcare associated infection were first consistently reported in England, and to end before widespread vaccination would complicate interpretation of results. Trusts that exclusively cared for children were excluded because of the distinct epidemiology of SARS-CoV-2 in children. The regression analysis was limited to the 96 of the 145 NHS acute hospital trusts where the more detailed data required for this analysis was available. All exclusion criteria were pre-established.
Replication	This was a retrospective analysis of national data from England. Code is provided to enable the analysis to be replicated using other national data, but we did not perform such replication as data were not available to us. To help interpret estimated regression coefficients we performed a series of simulation studies, generating synthetic transmission data-sets from a multitype branching process model, applying an observation model to obtain partially observed infection data, and replicating the above analysis.
Randomization	Randomization was not applicable as this was a retrospective analysis of national data and exposures of interest were not assigned to

Randomization	hospitals by the investigators, precluding randomisation.
Blinding	Blinding was not applicable as this was a retrospective analysis of national data and exposures of interest were not assigned to hospitals by investigators.

Reporting for specific materials, systems and methods

We require information from authors about some types of materials, experimental systems and methods used in many studies. Here, indicate whether each material, system or method listed is relevant to your study. If you are not sure if a list item applies to your research, read the appropriate section before selecting a response.

Materials & experimental systems

n/a	Involved in the study	
☒	☐	Antibodies
☒	☐	Eukaryotic cell lines
☒	☐	Palaeontology and archaeology
☒	☐	Animals and other organisms
☐	☒	Clinical data
☒	☐	Dual use research of concern

Methods

n/a	Involved in the study	
☒	☐	ChIP-seq
☒	☐	Flow cytometry
☒	☐	MRI-based neuroimaging

Clinical data

Policy information about clinical studies

All manuscripts should comply with the ICMJE guidelines for publication of clinical research and a completed CONSORT checklist must be included with all submissions.

Clinical trial registration	Not applicable: this was not a clinical trial.
Study protocol	Not applicable: this was retrospective analysis of national data
Data collection	Data come from nationally mandated reporting from all NHS acute care hospital trusts in England excluding only those that cared exclusively for children
Outcomes	Outcome measures (definite and probable healthcare associated infections) were defined based on ECDC criteria as described in the manuscript.
